# DDX54 Plays a Cancerous Role Through Activating P65 and AKT Signaling Pathway in Colorectal Cancer

**DOI:** 10.3389/fonc.2021.650360

**Published:** 2021-04-21

**Authors:** Yi Yu, Jing-Long Wang, Li-Li Meng, Chun-Ting Hu, Zhao-Wen Yan, Zhi-Ping He, Xiao-Qin Shi, Guo-Hui Fu, Li-Dong Zu

**Affiliations:** ^1^ Pathology Center, Shanghai General Hospital, Shanghai Jiao Tong University, Shanghai, China; ^2^ Department of Pathology, Shanghai Jiao Tong University School of Medicine, Shanghai, China

**Keywords:** DDX54, p65, AKT, proteomics, colorectal cancer

## Abstract

Colorectal cancer (CRC) is one of the most malignant cancers, and its incidence is still steadily increasing. The DDX RNA helicase family members have been found to play a role in various cancers; however, the role of DDX54 in colorectal cancer is still unclear and needed to be defined. Here, we found DDX54 was overexpressed in CRC tissues by the label-free mass spectrum, which was also verified in tissue microarray of colon cancer, as well as the CRC cell lines and TCGA database. High DDX54 level was correlated with tumor stage and distant metastasis, which always indicated a poor prognosis to the CRC patients. DDX54 could promote the proliferation and mobility of CRC cells through increasing the phosphorylation level p65 and AKT leading to the tumorigenesis. Here, we have preliminarily studied the function of DDX54 in CRC, which would improve our understanding of the underlying biology of CRC and provide the new insight that could be translated into novel therapeutic approaches.

## Introduction

Colorectal cancer (CRC) is a public health problem and is a major cause of morbidity and mortality worldwide as the third most common cancer and the second leading cause of cancer death (1). Although surgical resection is a relatively curative method for the treatment of colon cancer, metastasis and recurrence often occur after surgery, and the prognosis remains very poor (2, 3). Although extensive studies have demonstrated that CRC is a multi-factorial disease and frequently associated with physical, chemical, biological and genetic factors (4), and emerging evidence indicates that CRC is also a heterogeneous disease, arising through accumulation of genetic and epigenetic alterations, the etiology of CRC remains to be fully elucidated. Thus, it is important to identify the genes that contribute to CRC development.

DEAD-box proteins belong to the superfamily 2 (SF2) helicases (5). DEAD-box proteins are ATP dependent RNA helicases and include 38 members in humans (5). DEAD-box proteins function throughout the lifetime of cellular RNAs from synthesis to biological activity, and to inevitable decay (5). Recently, DEAD RNA helicase families were found to be expressed widely in the cancers including DDX5 in breast cancer (6) and lung cancer (7), DDX3 in prostate cancer (8) and hepatocellular cancer (9), and DDX27 in CRC (10) and gastric cancer (11). They are dysregulated in cancer in the form of involvement in chromosomal translocation, down-regulation, and over-expression of cancerous genes. DDX54, a member of the DEAD-box RNA helicase protein family, has been identified as a hormone-dependent interacting protein of estrogen receptors (ERs) (12) and CAR-binding protein (13). In addition, DDX54 also had a role on DNA damage repair (14) and myelination in oligodendrocytes (15). Recently, it was reported that the association of DDX54 to SNHG10 and PBX3 led to co-stability of each other and the complex could facilitate cell growth and motility in gastric cancer (16).

The important role of nuclear factor-κB (NF-κB) has been widely confirmed in various kinds of cancer (17). In colorectal cancer, NF-κB has a correlation with cancer-related processes involving the cell proliferation, apoptosis, angiogenesis, and metastasis (18). Targeting NF-κB pathway components may be a preventive measures and effective treatment approach to improve the patients’ overall outcome and quality of life. NF-κB is a heterodimer protein that consists of two subunits, p65 (RelA) and p50 which are required for activation and nuclear translocation of NF-κB. The promotive role of NF-κB in mediating colon carcinogenesis is illustrated by its regulation of a great diversity of genes positioned at the interface between cell proliferation, survival, and evasion from apoptosis. Mounting evidences support the role of NF-κB in regulating the expression of anti-apoptotic members of the Bcl-2 family like Bcl-2 and Bcl-xL (19). As the members of the IAP gene family, XIAP and Survivin have been identified as downstream targets of NF-κB signaling pathway (20). Regulation of NF-κB activity occurs at multiple levels. Hence, it is not simply adequate to understand the mechanism of nuclear localization and DNA binding of NF-κB subunits. Comprehending the molecular mechanism behind the regulation of each subunit of NF-κB complex and determination of their functionality will help to elucidate the NF-κB pathway as a whole.

The PI3K/AKT/mTOR pathway is another key regulator point of intracellular metabolism, cell growth and survival, angiogenesis, and invasiveness, which is involved in carcinogenesis and progression (21). This signaling pathway was verified to response to various intracellular and extracellular stimuli, such as inflammatory cytokines, growth factors and carcinogenic agents (22). AKT pathway is activated in a majority of human cancers due to mutations in PIK3CA or inactivation and decreased function of PTEN (23, 24).

In this study, we had screened the proteins with different expression level through proteomic analysis to found out the potential molecular marker. We found DDX54 was highly expressed in CRC tumor tissues compared to normal tissues. We also observed that CRC patients always had a relatively longer survival time with low DDX54 expression than those with high expression by colon cancer tissue microarray and prognosis prediction with online database. Moreover, DDX54 indeed inhibited the proliferation and metastasis in CRC cells. DDX54 might be a new molecular marker for CRC prognosis prediction.

## Materials and Methods

### Human Tissue Samples

Human colon cancer tissue samples and para-tumor tissue samples were obtained from the pathology center, Shanghai General Hospital/Faculty of Basic Medicine, Shanghai Jiao Tong University School of Medicine. These samples were immediately frozen in tubes and stored in liquid nitrogen after surgical resection. Written informed consent was obtained from each patient. Human CRC tissue samples for tissue microarray of 96 patients and corresponding adjacent specimens were obtained from patients who underwent colectomy or rectectomy at the Shanghai General Hospital (Shanghai, China) from October 2010 to August 2013. None of the patients were treated with immunotherapy, radiotherapy, or chemotherapy before surgery. The study was approved by the ethics committees of Shanghai Jiao Tong University, School of Medicine (Shanghai, China).

### Label-Free Mass Spectrometry Analysis

Sample was ground individually in liquid nitrogen and lysed with lysis buffer which containing 100 mM NH4HCO3 (pH 8), 6 M urea and 0.2% SDS, followed by 5 min of ultrasonication on ice. The lysate was centrifuged at 12,000*g* for 15 min at 4°C, and the supernatant was transferred to a clean tube. Extracts from each sample were reduced with 10 mM DTT for 1 h at 56°C, and subsequently alkylated with sufficient iodoacetamide for 1 h at room temperature in the dark. Then samples were completely mixed with four times volume of precooled acetone by vortexing and incubated at −20°C for at least 2 h. Samples were then centrifuged and the precipitation was collected. After washing twice with cold acetone, the pellet was dissolved by dissolution buffer, which contain 0.1 M triethylammonium bicarbonate (TEAB, pH 8.5) and 6 M urea. The LC-MS/MS spectra were collected and subjected to comparison with the UniProt human proteome database using Proteome Discoverer 1.4 (Thermo Fisher). Proteins were included for analysis when represented by at least two unique peptides. The raw MS data were processed using the MaxQuant software (version 1.5.3.8) (25) and matched to peptide sequences in the human UniProt protein database by the Andromeda algorithm (26). Relative, label-free quantification of the proteins was done using the MaxLFQ algorithm (27). The protein group files were imported into Perseus software (version 1.5.2.4) (28) to perform statistical analysis and validation. Hierarchical clustering was performed after z-score normalization of the data within Euclidean distance. Gene ontology (GO) analysis and KEGG analysis of differentially expressed proteins were generated by DAVID 6.8 (http://https://david.ncifcrf.gov/).

### Xenograft GC Nude Mice Model

Female athymic BALB/c nude mice (6–8 weeks old) were purchased from Shanghai Experimental Animal Center, Chinese Academy of Science. 5 × 10^6^ RKO cells infected with virus of siRNAs control and siRNAs targeted DDX54, were suspended in PBS and then subcutaneously injected into the nude mice. After two weeks, the tumors reached ~200 mm^3^. All mice were sacrificed after anesthesia and tumor size and weight were measured. Tumor volume was calculated, V = lengthwidth^2^/2 mm^3^. The experiments were approved by the animal research committee in Shanghai Jiao Tong University.

### Cell Culture

Human colorectal cancer cell lines were purchased from the Cell Bank of the Shanghai Institute for Biological Science (Shanghai, China). RKO, Caco2, SW620. SW480, HCT116, and DLD-1 were cultured in RPMI-1640 or high-glucose DMEM (Hyclone, Thermo Fisher, USA) medium supplemented with 10% fetal bovine serum (Hyclone) and 1% penicillin/streptomycin (Invitrogen, Carlsbad, CA, USA) at 37°C in a humidified atmosphere with 5% CO_2_. The other cells were cultured in DMEM with 10% FBS.

### Stable Cancer Cell Construction

DDX54 and shDDX54 virus stock solutions were purchased from Sangon Biotech (Shanghai, China). A day before virus infection, the colon cells were seeded into the six-well plate and grew well. The virus stock was diluted according to MOI (1:10 for v-DDX54 and 1:20 for v-siDDX54) and dropped into the wells along with polybrene addition. 24 h later, the diluted virus infected the cell again. Then the selective medium with antibiotics was placed. And after 2 weeks, the cells were cultured for assay.

### Quantitative Real-Time PCR (qRT-PCR)

Total RNA was extracted from cells or tissue samples after homogenization using Trizol reagent (15596-026, Invitrogen) according to the manufacturer’s instructions, and were reverse transcribed (RD0037A, Takara) into cDNA with specific RT primers. The relative mRNA levels were analyzed by qRT-PCR using a one-step SYBR PrimeScript™ RT-PCR Kit II (RR420A, Takara) in an ABI 7500 fast fluorescence temperature cycler. Glyceraldehyde 3-phosphate dehydrogenase (GAPDH) was chosen as the control for DDX54 normalization. The relative expression level of DDX54 was calculated using the 2^−ΔΔCt^ method after normalization. All experiments were repeated three times. The primer sequences were:

GAPDH-F  CTCCTCCTGTTCGACAGTCAGC

GAPDH-R  CCCAATACGACCAAATCCGTT

DDX54-F   TATGACAACAGCCTCAAGAT

DDX54-R   AGTCCTTCCACGATACCA

### Western Blotting

The whole cells were washed and lysed in RIPA buffer (89900, Thermo Scientific) supplemented with PMSF (phenylmethylsulfonyl fluoride) and protease inhibitors. Protein concentrations were determined with a BCA assay kit (Pierce, Rockford, IL, USA). Then the lysates were mixed with loading buffer, analyzed by 10% sodium dodecyl sulfate–polyacrylamide gel electrophoresis and then transferred onto polyvinylidene difluoride membranes (Millipore, Billerica, MA). After blocked with 5% skim milk in TBST at room temperature for 1 h, the membranes were incubated with different primary antibodies, including anti-DDX54 (1:1000, Proteintech, China), anti-P65, anti-p-P65, anti-AKT, anti-p-AKT, anti-mTOR, anti-p-mTOR, (1:1000, Cell Signaling Technology, Danvers, MA, USA), and anti-GAPDH (1:5000, Yeason, China) in 5% milk/TBST buffer at 4°C overnight, and then probed with horseradish peroxidase-conjugated anti-mouse or anti-rabbit IgG (1:5000, Jackson Immunoresearch Laboratories, West Grove, PA, USA) for 1 h. After washing three times with TBST, the membrane was developed with enhanced chemiluminescent plus substrate (Merck Millipore, Billerica, MA, USA), and the signal was recorded by Fluorchem E System (ProteinSimple, Santa Clara, CA, USA).

### *In Vitro* Cell Proliferation Assay

The cell proliferation assay was performed using a CCK-8 (Cell Counting Kit-8) kit (Dojindo, Japan). Colon cancer cells including RKO, Caco2, SW620 and HCT116 were seeded at a density of 2.5 × 10^3^ cells per well into 96-well plates with each well containing 200 μl medium. After culture for 24 h, the OD values were measured at 450 nm. The inhibitory agents’ treatments were carried every day 24 h after the cell seeding.

### Immunohistochemistry

Tumor specimens were fixed in 10% formalin overnight and embedded in paraffin. To observe DDX54 expression in CRC tissues, deparaffinized slides were treated with 3% H_2_O_2_ and subjected to antigen retrieval using 0.01 M citric buffer solution (pH 6.0). After overnight incubation with the rabbit anti-DDX54 antibody (1:100 dilution, Abnova) at 4°C, the slides were incubated for 15 min at room temperature with horseradish peroxidase-labeled polymer conjugated to a secondary antibody (Max Vision™Kit) and incubated with diaminobenizidine (DAB) for 2 min. The slides were then counterstained with hematoxylin. Appropriate positive and negative controls were tested in parallel. The IHC staining results were assessed by two pathologists without prior knowledge of patient data. The percentage of stained cells was scored as follows: 0% as 0; 1% to 10% as 1; 10% to 50% as 2; 51% to 80% as 3; 81% to 100% as 4, and the intensity of staining: no staining as 0; weak staining as 1; moderate staining as 2; strong staining as 3. Then multiplying the two was the score of each tissue (score range, 0–12) and the cutoff value was 6 to distinguish the high and low DDX54 staining.

### Statistical Analysis

Data are expressed as the mean ± standard deviation (SD). Student’s t-test was performed for continuous variables. The correlations of DDX54 staining and clinicopathological features were analyzed using the Pearson χ^2^ test. The survival analysis was carried out by the Kaplan-Meier method and evaluated using the log-rank test. The Cox proportional hazards regression model was used for univariate and multivariate analysis to evaluate the hazard ratio (HR) of prognostic factors. P < 0.05 was considered significant.

## Results

### MS Analysis in Colon Cancer

**Figure 1:**
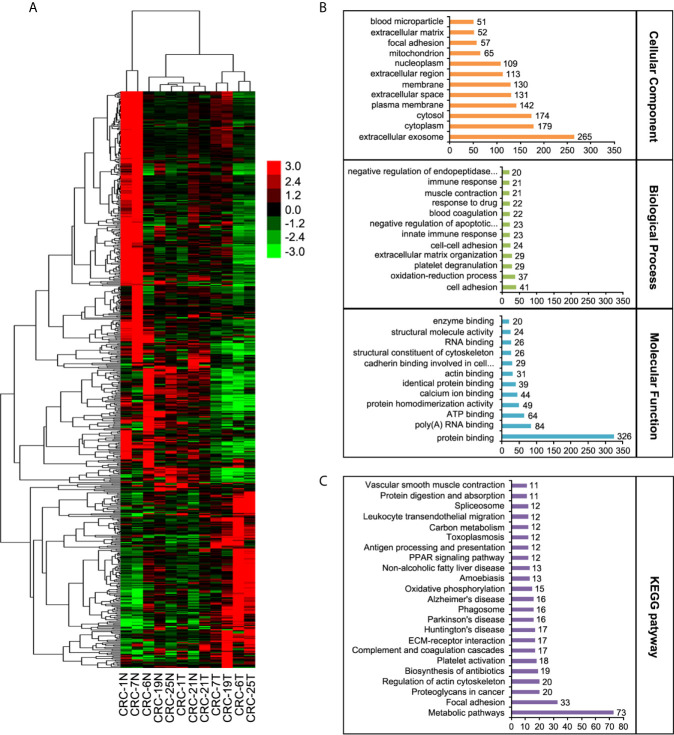
Label-free quantitative proteomic analyses. **(A)** The heat map of LC-MS/MS data analysis from the six CRC tumor tissues and their adjacent normal tissues. **(B)** Classification of differential proteins according to the GO analyses. **(C)** Classification of differential proteins according to the KEGG analyses.

In order to explore the mechanism which led to the tumorigenesis in colorectal cancer, we enrolled six pairs CRC patients and collected the tumor tissues and the adjacent normal tissues. The different protein expressions were analyzed between the tumor tissues and adjacent normal tissues through the mass spectrum (MS) technology. A total of 6847 proteins were identified from all tissue samples and 537 proteins were found to be differentially expressed (*p* < 0.05) (**Figure 1A**). 170 proteins were up-regulated and 163 proteins were down-regulated which displayed 1.5-fold quantitative alterations. According to the data from GO (gene ontology) database and KEGG (Kyoto encyclopedia of genes and genome) pathway database, 537 proteins were analyzed (**Figures 1B, C** **)**. KEGG analyses showed these proteins were most involved in metabolic pathways, focal adhesion, proteoglycans in cancer, and regulation of actin cytoskeleton, etc.

### Verification of MS in Colon Cells and Colon Cancer Patients

**Figure 2:**
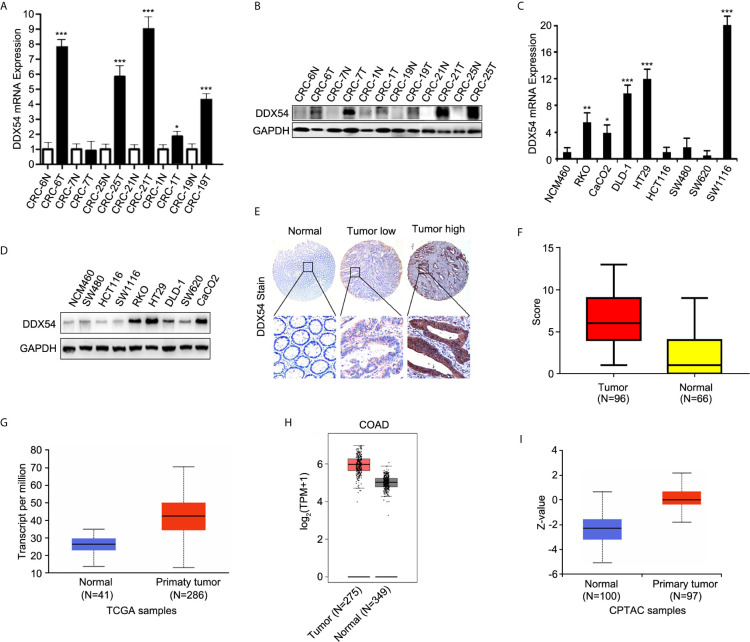
Validation of DDX54 from different expressive proteins. **(A)** Q-RT-PCR was performed to evaluate the level of DDX54 in CRC patients. GAPDH was used as a normalization control. The fold change was calculated using the 2^−ΔΔCt^ method. *, p < 0.05; ***, p < 0.001. All experiments were repeated three times. **(B)** DDX54 expression levels in CRC patients were examined through WB and GAPDH was loaded as the control. **(C)** Q-RT-PCR was carried out to evaluate the level of DDX54 in CRC cell lines. GAPDH was used as a normalization control. The fold change was calculated using the 2^−ΔΔCt^ method. *, p<0.05; **, p<0.01; ***, p<0.001. All experiments were repeated three times. **(D)** DDX54 protein levels were determined in CRC cell lines through WB and GAPDH was selected as the control. **(E)** The representative images of DDX54 staining by IHC. **(F)** The histogram of distribution for DDX54 staining score in CRC tissues and adjacent normal tissues. **(G)** DDX54 mRNA expression level was analyzed in colon adenocarcinoma from TCGA database at UALCAN website (http://ualcan.path.uab.edu/). **(H)** DDX54 mRNA expression level was analyzed in COAD (colon adenocarcinomas) from TCGA database at GEPIA website (http://gepia.cancer-pku.cn/). **(I)** DDX54 protein expression was analyzed in CRC patients of CPTAC (Clinical Proteomic Tumor Analysis Consortium) database. Z-values represent standard deviations from the median across samples for the given cancer type. Log2 Spectral count ratio values from CPTAC were first normalized within each sample profile, and then normalized across samples. (http://ualcan.path.uab.edu/).

In order to discover the differentially expressed proteins in colorectal cancer tissues compared to the adjacent benign tissues, four criterions were followed: 1) the gene should be highly expressed in the cancer tissues, 2) the up-regulated trend should be consistent in all patients, 3) the carcinogenic role of its similar proteins should be reported previously, 4) the result or the overexpressed gene should be verified in followed samples of the CRC patients and cell lines. Eventually, DDX54 was picked out to be investigated in our study. To confirm the alterations of protein expression revealed by quantitative proteomic analyses, DDX54 was determined by WB and qRT-PCR. The results were consistent with the quantitative proteomic analyses (**Figures 2A, B** **)**. The DDX54 expression was also detected in colorectal cancer cell lines and the results showed that DDX54 was significantly overexpressed in colon cancer cell lines compared to t he normal cell line NCM-460 (**Figures 2C, D** **)**.

Moreover, to further determine the expression status of DDX54 in CRC patients, immunohistochemistry staining was performed with tissue microarray containing 96 colon cancer tissues and 66 normal tissues (**Figure 2E**). And the staining score was calculated and summarized in **Figure 2F**. The correlation of DDX54 expression with clinicopathological characteristics of CRC patients was analyzed, the results determined by Chi-square test were shown in **Table 1**. High DDX54 expression was significantly associated with tumor stage and distant metastasis. There was no significant relation between DDX54 expression and other clinical parameters. Univariate and Multivariate Cox regression analysis both indicated that DDX54 expression was associated with the CRC distant metastasis and revealed that high DDX54 expression was an independent and favorable prognostic indicator for OS (**Tables 2**, **3**).

**Table 1:** Association between DDX54 expression and clinicopathological features of patients with colorectal cancer.

Clinicopathological parameters	Numbers of cases	DDX54 expression		χ^2^	*P* value
		Low	High		
ALL	96	47	49		
Age (years)				0.042	0.838
≥65	48	23	25		
<65	48	24	24		
Gender				0.058	0.809
Male	56	28	28		
Female	40	19	21		
Tumor size, cm				1.145	0.308
≥5	56	30	26		
<5	40	17	23		
T stage				2.851	0.415
T1	2	1	1		
T2	30	15	15		
T3	50	27	3		
T4	14	4	10		
Lymph node metastasis				2.52	0.284
N0	49	25	24		
N1	31	17	14		
N2	16	5	11		
Distant metastasis				6.139	0.013*
M0	90	47	43		
M1	6	0	6		
AJCC stage				8.015	0.044*
I	28	13	15		
II	18	12	16		
III	44	22	22		
IV	6	0	6		
Histological grade				1.309	0.253
H1-H3	78	33	45		
H4-H5	18	14	14		

*Statistically significant (P < 0.05).

**Table 2:** Univariate Cox regression analysis of potential prognostic factors for colorectal cancer patients.

Characteristics	n	HR	95% CI	P
Age (years)				
≥65	48			
<65	48	1.761	0.973–3.187	0.061
Gender				
Male	56			
Female	40	0.717	0.399–1.289	0.267
Tumor size, cm				
≥5	56			
<5	40	0.650	0.362–1.167	0.149
T stage				
T1+T2	32			
T3+T4	64	2.228	1.102–4.503	0.026*
Lymph node metastasis				
N0	49			
N1+N2	47	1.535	0.852–2.765	0.154
Distant metastasis				
M0	90			
M1	6	15.537	5.67–42.576	<0.001***
AJCC stage				
I, II	46			
III, IV	50	1.963	1.073–3.591	0.029
Histological grade				
H1-H3	78			
H4-H5	18	0.614	0.341–1.107	0.105
DDX54 expression				
Low	47			
High	49	3.128	1.634–5.988	0.001**

Statistically significant (*P < 0.05) **P < 0.01, ***P < 0.001.

**Table 3:** Multivariate Cox regression analysis of potential prognostic factors for colorectal cancer patients.

Characteristics	HR	95%CI	P
Tumor size, cm	0.515	0.266–0.998	0.049
T stage	1.714	0.714–4.112	0.228
Lymph node metastasis	1.750	0.326–9.403	0.514
Distant metastasis	13.737	3.456–54.6	<0.001***
AJCC stage	0.742	0.11–5.031	0.760
Histological grade	0.696	0.367–1.321	0.268
DDX54 expression	2.485	1.250–4.943	0.009**

**P < 0.01, ***P< 0.001.

This result was further confirmed at the online websites involving the TCGA database. DDX54 was highly expressed in CRC patients than normal patients in Ualcan and GEPIA website (**Figures 2G, H** **)** at mRNA level. In human protein atlas, as the same as the mRNA level, DDX54 protein level was also up-regulated in CRC patients in spite of the stage (**Figure 2I**). Taken together, DDX54 was indeed up-regulated in CRC cells and might have an ontogenetic role in CRC.

### High DDX54 Level Indicated a Poor Prognosis

**Figure 3:**
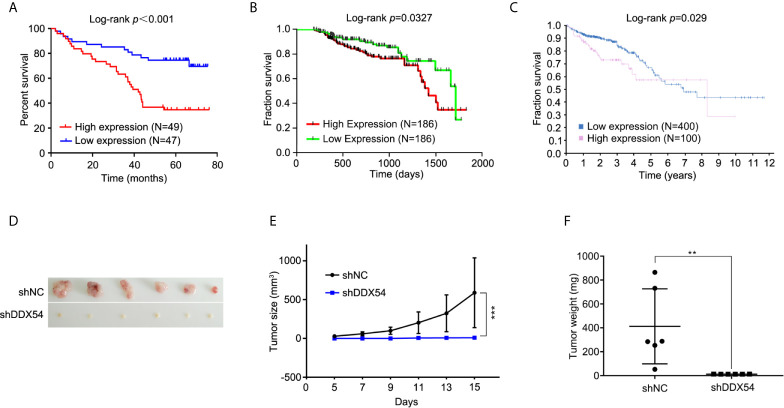
DDX54 expression and significance in colorectal cancer was analyzed in TCGA database. **(A)** The relation between DDX54 mRNA level and CRC survival time was analyzed of tissue microarray. **(B)** Kaplan-Meier plots of the overall survival of patients stratified by DDX54 expression were analyzed on CRC patients from TCGA database with the less than 5-followup period. **(C)** The relation between DDX54 mRNA level and survival was analyzed on CRC patients from the Human Protein Atlas. FPKM, Fragments Per Kilobase per Million. **(D)** The representative images for subcutaneous tumors of DDX54 knockdown mice and relative controls. **(E)** The size of subcutaneous tumors was calculated at different times, ***P < 0.001. **(F)** The tumor weights of mice with control or DDX54 knockdown injection were counted, **P < 0.01.

To evaluate the association between the DDX54 expression and prognosis of CRC, Kaplan–Meier survival curves were made and analyzed. DDX54 expression was significantly associated with the overall survival time by staining score of tissue microarray (**Figure 3A**). Moreover, this association between DDX54 expression and survival was also performed in CRC patients with 5 years follow-up time from TCGA database (**Figure 3B**). The further confirm was also obtained from the human protein atlas website (**Figure 3C**). These results indicated that the CRC patients with high DDX54 expression had a greatly poorer overall survival time than those with low DDX54 expression. In consideration of the important role of DDX54 on CRC, the nude mice were subcutaneously injected with RKO cells with v-shDDX54 infection. The subcutaneous tumors with different volume were shown in **Figure 3D**, which showed that DDX54 knockdown significantly inhibited the growth of CRC cells. The tumor growth curve was calculated at different time (**Figure 3E**) and the tumor weight was also determined in **Figure 3F**.

### The Function of DDX54 Was Determined in CRC Cells

**Figure 4:**
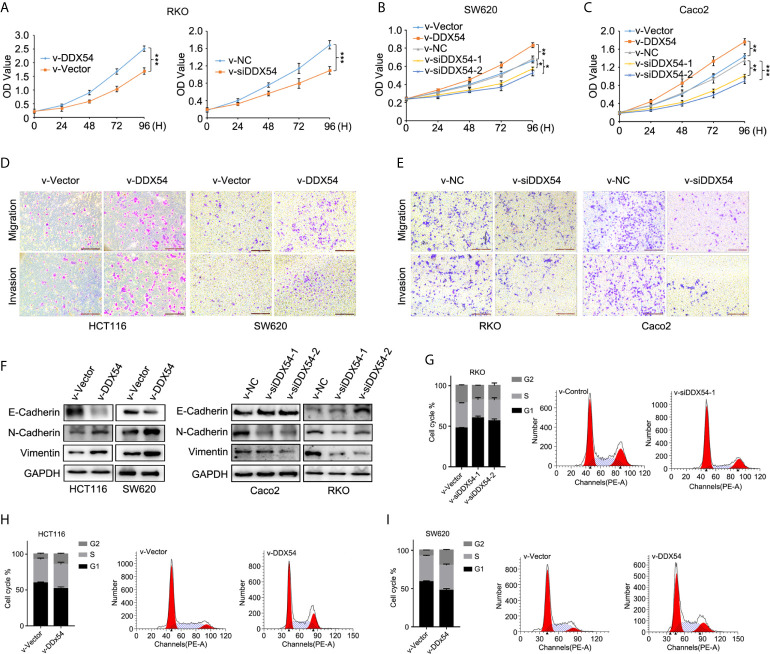
DDX54 had a role on CRC cells. **(A)** The proliferative effects of DDX54 were examined through CCK8 assay in RKO stable cells, ***P < 0.001. **(B)** The role of DDX54 on proliferation was detected in SW620 cells, *P < 0.05, **P < 0.01. **(C)** DDX54 promoted the proliferation in Caco2 cells, **P < 0.01, ***P < 0.001. **(D)** The migration and invasion ability of HCT116 and SW620 was promoted by DDX54. **(E)** DDX54 knockdown attenuated the metastatic ability of RKO and Caco2. **(F)** EMT markers were determined by WB in CRC cells with DDX54 overexpression or knockdown. **(G–I)** DDX54 promoted the cell cycle process in CRC cells.

In order to evaluate the function of DDX54 in CRC, we constructed the stable RKO cell line with DDX54 overexpression or DDX54 knockdown, respectively. Then the proliferative ability of these RKO cells was determined through CCK8 assay. The results showed that the RKO cells with DDX54 overexpression grew faster than those with normal DDX54. Conversely, the RKO cells with DDX54 knockdown grew slower than those normal cells (**Figure 4A**). The similar results of DDX54 on the proliferation were further verified in SW620 (**Figure 4B**) and Caco2 (**Figure 4C**). The ability of tumor metastasis was further determined by the Transwell experiments and the results showed that DDX54 promoted the mobility of HCT116 and SW620 cells (**Figure 4D**). Conversely, DDX54 knockdown inhibited the migration and invasion ability in RKO and Caco2 cells (**Figure 4E**). EMT was considered as a major reason for tumor mobility which was characterized by down-regulation of epithelial markers (e.g., E-cadherin) and up-regulation of mesenchymal markers (e.g., Vimentin and N-cadherin) (29). In this respect, we found that E-cadherin was decreased and N-cadherin and Vimentin were up-regulated by enhanced DDX54, then the opposite effect was observed by knockdown DDX54 (**Figure 4F**). E-cadherin expression was markedly increased, and N-canherin and Vimentin expression were greatly reduced in RKO cells with DDX54 knockdown compared with the control by IHC (**Supplementary Figure 1**), suggesting that DDX54 promoted CRC metastasis is potentially responsible for EMT process. Moreover, the effect of DDX54 on cell cycle was also detected, and the results showed that the cells with DDX54 knockdown were subjected to be arrested at of G1 stage (**Figure 4G**). In contrary, CRC cells with enhanced DDX54 were not subjected to be arrested at G1 stage (**Figures 4H, I** **)**.

### Overexpressed DDX54 Activated the P65 and AKT Pathway

**Figure 5:**
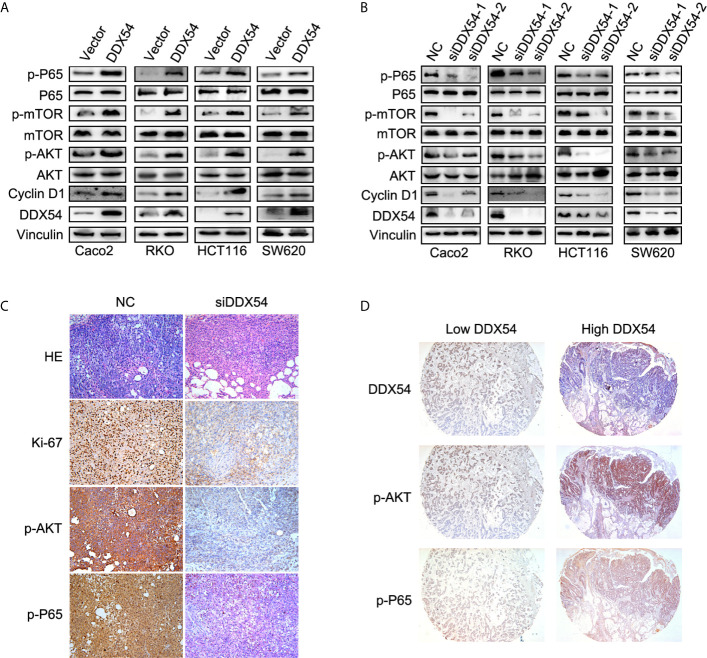
DDX54 activated the NF-κb and AKT/mTOR pathway. **(A)** The enhanced DDX54 up-regulated the phosphorylation level of P65 and AKT in RKO, Caco2, SW620, and HCT116 cells. **(B)** The phosphorylation level of P65 and AKT was reduced by DDX54 inhibition with RNAi in RKO, Caco2, SW620, and HCT116 cells. **(C)** The Ki-67, p-P65, and p-AKT were examined in mice subcutaneous tumors with DDX54 knockdown. **(D)** The level of p-P65 and p-AKT in CRC patients with high DDX54 was higher than those with low DDX54.

We further examined the effects of altered DDX54 on the activities of the pathways in CRC cells. We found that only the p65 and AKT was phosphorylation level obviously elevated while the others were the same as the controls in Caco2, RKO HCT116 and SW620 cells (**Figure 5A**). And reversely, knockdown DDX54 by RNAi conferred the reduction of phosphorylation level of p65 and AKT in these CRC cells (**Figure 5B**). Furthermore, the same role of DDX54 knockdown on the repressing p-p65 and p-AKT was also observed in nude mice model through immunochemical staining (**Figure 5C**). Meanwhile, Ki67 staining was also detected and showed that DDX54 knockdown inhibited the CRC cell proliferation which was consistent with the Cyclin D1 status regulated by DDX54 (**Figures 5A, B** **)**. In CRC patients, high expressed DDX54 always showed high phosphorylation level of p65 and AKT (**Figure 5D**). Taken together, DDX54 regulated the cell proliferation and metastasis through activating P65 and AKT pathway.

**Figure 6:**
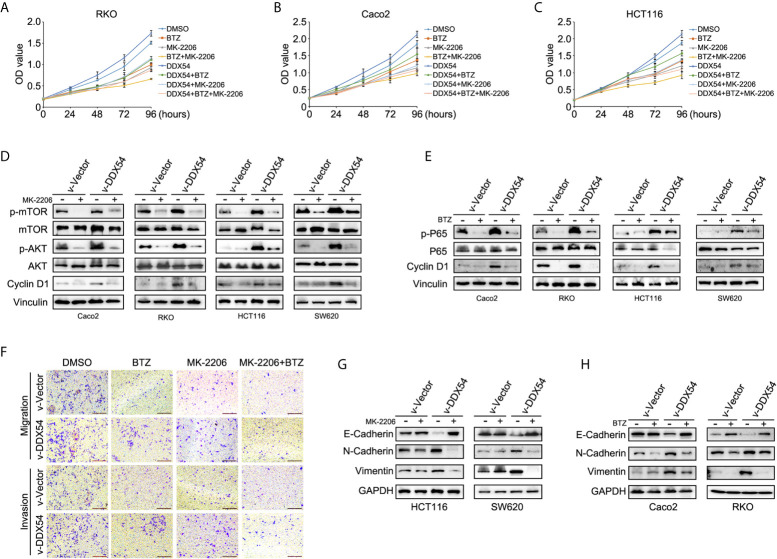
Inhibition of NF-κb and AKT/mTOR pathway weakened the carcinogenic effects of CRC cells by DDX54. **(A–C)** BTZ (Bortezomib, 2 nM) and MK2206 (1 μM) (inhibitor of NF-κb and AKT pathway respectively, Selleck) repressed the proliferative role of DDX54 on CRC cells. **(D, E)** The phosphorylation level of P65 and AKT was determined in CRC cells with different treatments of DDX54 and inhibitors. **(F)** The metastasis ability was weakened by inhibitors. **(G, H)** EMT markers were detected in CRC cells with DDX54 and inhibitor treatments.

To examine whether DDX54 promoted CRC progression through p65 and AKT pathway, the CRC cells were treated with Bortezomib (Btz, p-p65 inhibitors) and MK-2206 (AKT inhibitors) respectively. The results showed that BTZ and MK-2206 could inhibit the cell growth respectively and neutralized the promotive effect of DDX54 on cell growth in RKO, Caco2 and HCT116 cells (**Figures 6A–C**). Moreover, the inhibitors could repressed the phosphorylation level of P65 and AKT, respectively, and this inhibitory effect was attenuated by enhanced DDX54 (**Figures 6D, E** **)**. In addition, the metastatic ability contributed by DDX54 was also inhibited by the inhibitors of P65 and AKT pathways (**Figure 6F**). The EMT markers were also detected in condition of BTZ and MK2206 treatment. The results showed that E-cadherin was up-regulated by BTZ and MK-2206–treated cells with enhanced DDX54 while N-cadherin and Vimentin was down-regulated respectively compared to only DDX54 transfected cells (**Figures 6G, H** **)**.

## Discussion

In recent years, much attention has been paid to how to improve the diagnosis and prognosis of CRC, although the great improvements have been made, the 5-year OS rate of CRC patient remains still low. Benefits from the genome sequencing, the attentions had been turned back to how to predict the cancer and the benefits from the targeted therapy. The gene functions involved in etiology of CRC had been widely investigated. In current study, we aimed to find the biomarker gene to distinguish the tumor from normal tissues. We performed the proteomic analysis in CRC patients by LC-MS, and we found 573 different proteins. Of which, DDX54 is confirmed to be highly expressed in CRC tumor tissues (**Figure 2**), which implied a closely association of DDX54 with colon cancers. Recently, increasing publications about DDX RNA helicase family members have been described an important role on CRC development. DDX5 can bind to Lnc-NEAT1 to promote its stability and this complex can sequentially activated Wnt signaling (30). In another research, the authors found p68, also named as DDX5, and β-catenin increased RelA mRNA and protein expression. DDX5 was perceived to associate with RelA promoter while β-catenin at the TCF4/LEF (TBE) sites thereby potentiating RelA transcription (31). DDX27 was also observed to be highly expressed in CRC tissues and increased the proliferation and motility through promote p65 to bind to the promoter region of its target genes (10). On the other hand, highly expressed DDX27 was found to promote promotes sensitivity to 5-fluorouracil (32). Moreover, DDX3 and DDX1 were also found to have an oncogenic role on CRC (33, 34).

Furthermore, to investigate the underlying mechanism for DDX54 to promote CRC progression, we showed that DDX54 had a promotive effect on CRC cell proliferation and metastasis through activating NF-κb and AKT-mTOR signaling pathway (**Figure 5**). When we blocked the NF-κb and AKT-mTOR signaling pathway with BTZ and MK-2206, the cell growth and mobility were both inhibited. In DDX54 overexpressed cells, the effect on proliferation and metastasis of inhibitors was reversed to the primary level (**Figure 6**). Here, we found that the concurrent activation of NF-κb and AKT pathway was necessary for the role of DDX54 in CRC. The NF-κb and AKT pathway were both activated in some certain cancers due to unknown mechanism by which the cancer cells had some selective benefits (35, 36). In worth to date that inhibiting either pathway alone had partial effects on tumor proliferation and metastasis (37). One reasonable explanation is that these two pathways activate a same set of downstream targets. If so, inhibition of either alone would be insufficient to block these targets.

In the current assay, we also indicated the association between the high expressed level DDX54 and the prognosis of CRC patients. By tissue microarray, we found that the CRC patients with overexpressed DDX54 always had the poor survival time compared to those with lower DDX54. Since DDX54 belong to the RNA helicase family, regulated a set of genes which participated in the important cell process. In summary, we found the high level DDX54 was associated with the tumorigenesis. DDX54 could promote the proliferation and motility through elevating the p-p65 and p-AKT level.

## Data Availability Statement

The data sets presented in this study can be found in online repositories. The names of the repository/repositories and accession number(s) can be found in the article/**Supplementary Material**.

## Ethics Statement

The studies involving human participants were reviewed and approved by the ethics committees of Shanghai Jiao Tong University School of Medicine. The patients/participants provided their written informed consent to participate in this study. The animal study was reviewed and approved by the ethics committees of Shanghai Jiao Tong University School of Medicine. The animal study was reviewed and approved by the ethics committees of Shanghai Jiao Tong University School of Medicine.

## Author Contributions

L-DZ, G-HF, and YY conceived and designed the research. YY, J-LW, L-LM performed the experiments and analyzed the data, C-TH, X-QS contributed to collect clinical samples, Z-PH, Z-WY conducted the IHC. All authors have made a substantial, direct, and intellectual contribution to the work and approved it for publication.

## Funding

This work was supported in part by First Round of 3-year Action Plan to promote clinical skills and clinical innovation in Municipal Hospitals of Shanghai (16CR2039B), Shanghai Municipal Key Clinical Specialty Construction Project (NOshslczdzk01303).

## Conflict of Interest

The authors declare that the research was conducted in the absence of any commercial or financial relationships that could be construed as a potential conflict of interest.
