# Supplementary material for: DDX54 Plays a Cancerous Role Through Activating P65 and AKT Signaling Pathway in Colorectal Cancer
Source: Front Oncol. 2021 Apr 21;11:650360. doi: 10.3389/fonc.2021.650360 (PMC8097168; doi:10.3389/fonc.2021.650360)
Supplement: Supplementary file 1 [file DataSheet_1.docx]

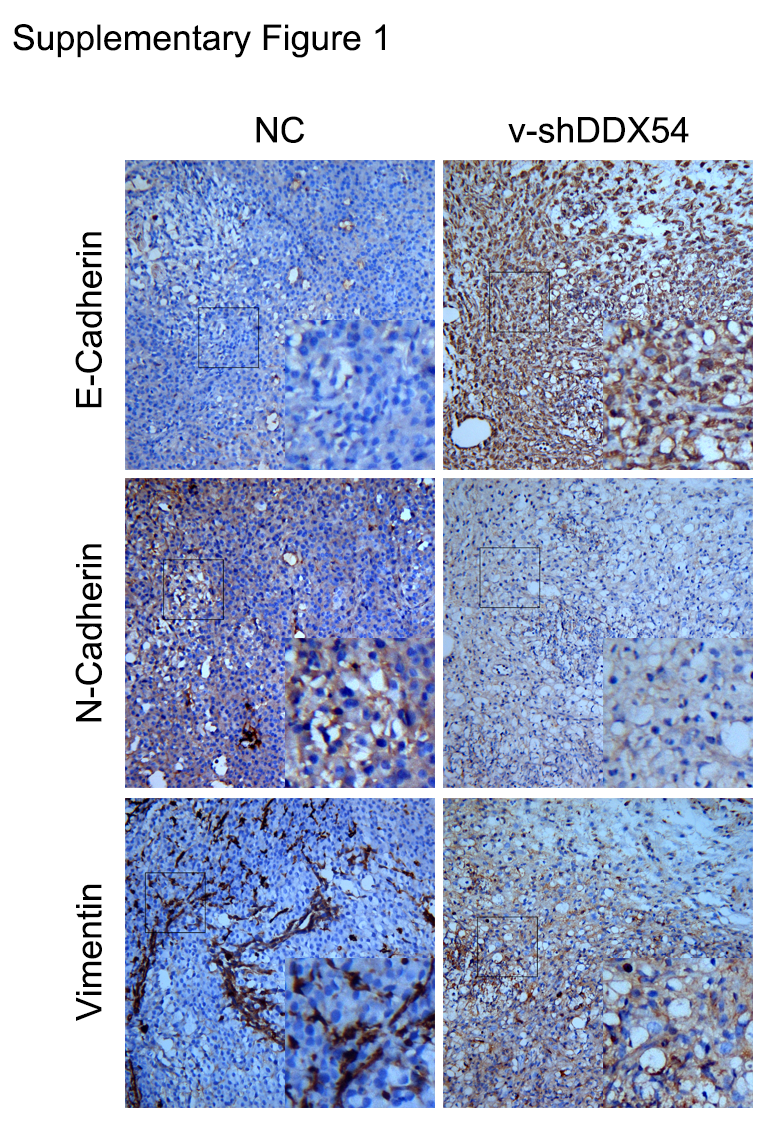


The E-Cadherin, N-cadherin, Vimentin were examined in mice subcutaneous tumors with DDX54 knockdown.

**Supplementary MS data**

The names of the repository/repositories is [https://www.iprox.org/, and](https://www.iprox.org/,%20and) project ID is IPX0002855000
